# Solution structures of the *Bacillus cereus* metallo-β-lactamase BcII and its complex with the broad spectrum inhibitor *R*-thiomandelic acid

**DOI:** 10.1042/BJ20131003

**Published:** 2013-11-22

**Authors:** Andreas Ioannis Karsisiotis, Christian F. Damblon, Gordon C. K. Roberts

**Affiliations:** *The Henry Wellcome Laboratories of Structural Biology, Department of Biochemistry, University of Leicester, Leicester LE1 9HN, U.K.; †The School of Pharmacy and Pharmaceutical Sciences, Biomedical Sciences Research Institute, University of Ulster, Coleraine BT52 1SA, Northern Ireland, U.K.; ‡Chimie Biologique Structurale, Institut de Chimie, Université de Liège, 4000 Liège, Belgium

**Keywords:** antibiotic resistance, enzyme structure, inhibitor binding, metallo-β-lactamase, NMR spectroscopy, zinc enzyme, HSQC, heteronuclear single-quantum coherence, MBL, metallo-β-lactamase, NOE, nuclear Overhauser effect

## Abstract

Metallo-β-lactamases, enzymes which inactivate β-lactam antibiotics, are of increasing biological and clinical significance as a source of antibiotic resistance in pathogenic bacteria. In the present study we describe the high-resolution solution NMR structures of the *Bacillus cereus* metallo-β-lactamase BcII and of its complex with *R*-thiomandelic acid, a broad-spectrum inhibitor of metallo-β-lactamases. This is the first reported solution structure of any metallo-β-lactamase. There are differences between the solution structure of the free enzyme and previously reported crystal structures in the loops flanking the active site, which are important for substrate and inhibitor binding and catalysis. The binding of *R*-thiomandelic acid and the roles of active-site residues are defined in detail. Changes in the enzyme structure upon inhibitor binding clarify the role of the mobile β3–β4 loop. Comparisons with other metallo-β-lactamases highlight the roles of individual amino-acid residues in the active site and the β3–β4 loop in inhibitor binding and provide information on the basis of structure–activity relationships among metallo-β-lactamase inhibitors.

## Abstract 2

Metallo-β-lactamases are important in antibiotic resistance in micro-organisms. We report the first solution structure of a metallo-β-lactamase and its complex with an inhibitor, allowing the key flexible loops flanking the active site and their role in inhibitor binding to be properly defined.

## INTRODUCTION

The rapid increase in resistance to β-lactam antibiotics is a major clinical and public health concern, as these antibiotics have long been key to the treatment of serious bacterial infections. The β-lactamases, enzymes which inactivate β-lactam antibiotics by hydrolysis of their endocyclic β-lactam bond, are a major source of this resistance [1]. In the sequence-based Ambler classification of β-lactamases, classes A, C and D are serine enzymes, whereas class B represents the zinc-dependent MBLs (metallo-β-lactamases) [2,3]; the latter are of particular concern since they are located on highly transmissible plasmids and have a broad spectrum of activity against almost all β-lactam antibiotics. MBLs are grouped according to sequence similarities and zinc co-ordination into three subclasses, B1, B2 and B3 [2,4,5]. Subclasses B1 and B3, operating with one or two zinc atoms, are broad-spectrum enzymes acting on almost all β-lactam substrates apart from monobactams, whereas subclass B2 enzymes, operating with one zinc atom, are strict carbapenemases acting poorly on penicillins and cephalosporins. MBLs in all three subclasses give rise to clinical problems [6,7]. The VIM and IMP families of transferable subclass B1 MBLs have a worldwide spread and are encountered in several Gram-negative pathogenic bacteria. In addition, the occurrence of new transferable MBLs such as NDM-1 [8] is an indicator of a serious, evolving and escalating problem.

A number of X-ray crystal structures of MBLs have been determined, a list of which can be found on the MBLED (Metallo-Beta-Lactamase Engineering Database; http://www.mbled.uni-stuttgart.de/) [5], mostly of enzymes of the ubiquitous and clinically relevant B1 subclass such as BcII, CcrA, IMP-1, BlaB, SPM-1, VIM-2 and NDM-1, but also including structures of members of the B2 and B3 subclasses. All known MBL structures share the same topology, an αβ/βα sandwich fold; this so-called MBL fold defines a rapidly expanding family of zinc metallo-hydrolases [10].

By contrast with the success in developing inhibitors of serine β-lactamases such as clavulanic acid, no clinically efficient inhibitors of MBLs have yet been reported [11,12]. Whereas all MBLs share conserved motifs, the sequence similarity is low and the range of active site architectures makes the development of broad spectrum inhibitors difficult. Crystal structures have been determined for complexes of MBLs with several different inhibitors [13–20], but although the *Bacillus cereus* MBL BcII is the most extensively studied MBL, no structure for an inhibitor complex of this enzyme has hitherto been reported.

It is clear that the most promising inhibitors directly co-ordinate one or both zinc atoms through, for example, thiol [21], carboxylate [17], tetrazole [13] or hydrazone [22] groups, and thiol-containing compounds show activity as broad-spectrum MBL inhibitors [20,21,23]. The potency of the thiol plus carboxylate combination is well established; even the simplest mercaptocarboxylate compounds, such as mercaptoacetic acid and 2-mercaptopropionic acid, are potent inhibitors of subclass B1 MBLs ([24–26] and S. Kumar, A.I. Karsisiotis, C. F. Damblon and G.C.K. Roberts, unpublished work). We have previously identified *R*-thiomandelate as a promising reasonably broad-spectrum inhibitor of MBLs, combining a thiol functionality which binds simultaneously to the two zinc atoms with a carboxylate function which increases its inhibitory potency [21,27]. We now report the solution structures of *B. cereus* BcII, a representative member of the important subclass B1 of the MBL family, alone and in its complex with *R*-thiomandelate. Analysis of the structures throws new light on the function of the much-discussed ‘mobile loop’ or ‘flap’ (the β3–β4 loop), which is conserved in B1 MBLs and plays a role in inhibitor binding and catalytic activity [3,14,16,17,28–30], and on the enzyme–ligand interactions which are likely to be important for inhibitor design.

## EXPERIMENTAL

### Protein production and purification

Singly (^15^N) and doubly (^13^C, ^15^N) labelled samples of BcII and the BcII–thiomandelate complex were produced as described previously [31].

### NMR spectroscopy and sequence-specific assignments

All NMR experiments were performed at 308 K in 20 mM Mes, pH 6.4, containing 10% ^2^H_2_O, 100 mM NaCl and 0.2 mM ZnCl_2_. Backbone and side chain sequence-specific assignments were obtained using standard triple resonance and TOCSY experiments as described prevously [31]. NOEs (nuclear Overhauser effects) were assigned in ^15^N-edited [32,33] and ^13^C-edited [34] NOESY-HSQC (heteronuclear single-quantum coherence) spectra, acquired at 600 MHz (cryoprobe) and 800 MHz (cryoprobe) respectively, with a mixing time of 80 ms. A ^13^C-edited NOESY-HSQC [34] spectrum at 800 MHz, optimized for the aromatic region, was used for obtaining both side chain assignments of the aromatic residues and NOE constraints.

^13^C-filtered experiments [35] (with ^13^C-protein and natural abundance ligand) provided unambiguous assignments of the resonances of the CαH (benzylic proton; 5.223 p.p.m.) and the *ortho* aromatic protons (6.941 p.p.m.) of the bound thiomandelate. Both shifts were verified by a series of double-filtered experiments. The observation that the two *ortho* protons are magnetically equivalent indicates that the aromatic ring of bound thiomandelate can ‘flip’ rapidly by 180°, as is commonly observed for phenylalanine and tyrosine residues in proteins [36] and for aromatic rings of bound ligands (e.g. [37]). The chemical shifts of the *meta*- and *para*- protons of the aromatic ring of the inhibitor appeared to be degenerate.

### Structural calculations and automated NOE assignments

Automated NOE assignments and structure calculations were performed using the CANDID module [38] and the torsion angle dynamics algorithm CYANA 2.1 [39] using default calibration parameters and optimized chemical shift tolerance windows. All NOESY peaks were picked using the peak-picking mode in Sparky (http://www.cgl.ucsf.edu/home/sparky) for individual strips with noise and artefact peaks removed manually. Peak lists in the DYANA/XEASY format were prepared using Sparky. The finalized peak lists used for the structural calculations before refinement were produced through several rounds of manual peak checks and automated unbiased NOE assignment runs using the CANDID module, aiming for optimum cycle1 and final CYANA target function values, maximum assignment percentages and numbers of generated constraints and improved structural parameters. This iterative process produced better constraint sets and, through the manual inspection of the NOESY spectrum, resolved missing or incomplete assignments. Ligand–protein distance constraints were obtained from experiments, using ^13^C-protein and natural abundance ligand, designed to detect NOEs between protons bound to ^13^C and protons not bound to ^13^C. Out of the 13 NOE-derived constraints obtained, four were assigned to the CαH of *R*-thiomandelate. The remaining nine constraints were assigned to one of the pseudoatoms (QG for the *ortho* protons, QD for the *meta* protons and QR for all the ring protons) of the thiomandelate ring on the basis of criteria including chemical shifts, probable distance in preliminary structural calculations and relative intensities of the corresponding peaks. Cross-peak heights were used for NOE distance calibration. Hydrogen bond restraints within secondary-structure elements were identified by the first no-violations refinement run. Only those observed in over 75% of the calculated structures were considered and were cross-validated with deuterium exchange experiments prior to introduction into the refinement. Automated stereospecific assignments from CYANA were introduced at the start of refinement. As discussed in the Supplementary Online Data and Table S1 (at http://www.biochemj.org/bj/456/bj4560397add.htm), the structures were calculated both with and without dihedral angle constraints obtained from the TALOS [41] database and we focus on the structures obtained without dihedral constraints; Table 1 gives the structural statistics for the structures calculated without TALOS constraints and Supplementary Table S2 (at http://www.biochemj.org/bj/456/bj4560397add.htm) gives these statistics for structures calculated with these constraints. The structures calculated with and without dihedral angle constraints had very similar RMSD values (0.35–0.38 for backbone atoms) and similar Ramachandran statistics (>98% of residues in the core and allowed regions), but the structures calculated with dihedral angle constraints showed significantly more distance and angle violations.

**Table 1:** Structural statistics and agreement with experimental data for the refined structures of BcII and the BcII–*R*-thiomandelate complex Target function, RMSD and energy values are calculated for a bundle of 20 structures unless specified otherwise. RTM, *R*-thiomandelate.

Parameter	Statistics	Free BcII	Complex
CYANA / CANDID	Input NOE peaks	13088	12499
	Unassigned peaks	1071	1131
	Assignment percentage	91.8	91.0
	Target function (cycle1)	247.9	204.92
	Target function (final)	7.03	3.71
RMSD, Å (residues 7–227) cycle1	Backbone/heavy atoms	1.07/1.53	1.32/1.75
RMSD, Å (residues 7–227) final	Backbone/heavy atoms	0.30/0.67	0.36/0.72
NOE constraints	Upper distance limits	7190	6578
	Short range (*i*=1)	3001	2797
	Medium range (1<*i*≤4)	1277	1059
	Long range (*i*>4)	2912	2722
	Removed during refinement	9	2
Other constraints	Zinc distance constraints	7	9
	RTM constraints	−	13
	Dihedral restraints*	0	0
	Hydrogen bonds	42	36
	Stereospecific assignments	143	109
Target function (refinement)	Minimum	3.34	2.22
	Average (20 structures)	4.3±0.4	2.4±0.1
	Average (50 structures)	4.9±0.6	2.6±0.2
RMSD of experimental restraints (20 structures)	Upper distance limits (Å)	0.007±0.001	0.004±0.0002
	Lower distance limits (Å)	0.002±0.002	0.002±0.001
	Torsion angles (°)	0.01±0.01	0.008±0.016
Van der Waals energy sums	20 structures	19±1	11.1±0.6
	50 structures	21±2	12±1
Mean violations (20/50 structures)	Distance >0.5 Å	0/0	0/0.02±0.14
	Angle >5°	0/0.2±0.6	0/0
	Van der Waals	0/0	0/0
RMSD, Å (residues 7–227) final structures	Backbone/heavy atoms	0.35/0.72	0.38/0.73
Ramachandran statistics (CANDID stage/refinement)	Core (%)	71.3/70.4	70.6/70.8
	Allowed (%)	27.2/27.9	28.6/27.6
	Generously allowed (%)	1.0/1.0	0.3/0.8
	Disallowed (%)	0.5/0.6	0.5/0.8

*The structures were calculated with no external (TALOS) dihedral angle input. (The statistics for structures calculated with inclusion of the dihedral angle constraints are given in Supplementary Table S2 at http://www.biochemj.org/bj/456/bj4560397add.htm.)

### Structure refinement and validation

CYANA/CANDID distance constraints were used directly in structure refinement since the final calculation filters out ambiguous distance constraints. The two metal atoms and, in the case of the complex, the inhibitor molecule, were incorporated through the use of the generic linker system present in CYANA as described in the Supplementary Online Data. The refinement protocol used consisted of five cycles of simulated annealing with a high start temperature (8000 K) and a gradual slow cooling to a low temperature (100 K) in a large number of steps (20000). This standard torsion angle-based simulating annealing protocol with all constraints applied simultaneously is combined with cycles of redundant dihedral angle constraints (REDAC; [42]). Several cycles of this combined refinement protocol, which eliminates violations and progressively reduces target function values while improving local and overall quality, were used to generate the final converged structures. Out of an ensemble of 100 converged structures generated, the 20 best (lowest CYANA target function during refinement) structures were selected. Structure validation was carried out using PROCHECK-NMR [43], WHAT-IF [44] and the iCING (https://lamp-lbi-43.rcs.le.ac.uk/icing/#welcome) web interface. All molecular models were viewed and assessed using MOLMOL [45], which was used for all RMSD calculations and comparisons (typically from the bundle of the 20 lowest energy structures). All distance restraint distribution plots are based on the CYANA macro ‘dcostat’.

### Databank accession codes

The complete ^1^H, ^13^C and ^15^N chemical shifts and NMR restraints have been deposited in the BMRB (BioMagResBank) under accession codes 19047 (BcII) and 19048 (BcII–*R*-thiomandelic acid) and co-ordinates have been deposited in the PDB under accession codes 2M5C (BcII) and 2M5D (BcII–*R*-thiomandelic acid).

## RESULTS AND DISCUSSION

### Resonance assignments and structure calculations

The strategy for obtaining the sequence-specific assignments has been described previously [31]. ^1^H, ^13^C and ^15^N resonances were assigned to over 96% completion for both free BcII and the BcII–inhibitor complex. Automated NOE assignments and structure calculations were performed using the CANDID/CYANA protocol as described in the Experimental section. The CANDID stage of the calculations yielded 91.8% and 91.0% assignment of all NOE peaks for the free enzyme and complex respectively. The solution structures were calculated using 7190 (~31 per residue) non-ambiguous NOE distance restraints for the free enzyme and 6578 (~29 per residue) for the complex with *R*-thiomandelate. All structures were refined to the point where there were no angle violations larger than 5° and no distance violations larger than 0.2 Å (1 Å=0.1 nm) in 80% of the structures. The key statistics for the structure calculations are presented in Table 1.

### The solution structure of the free enzyme: comparison with crystal structures

**Figure 1:**
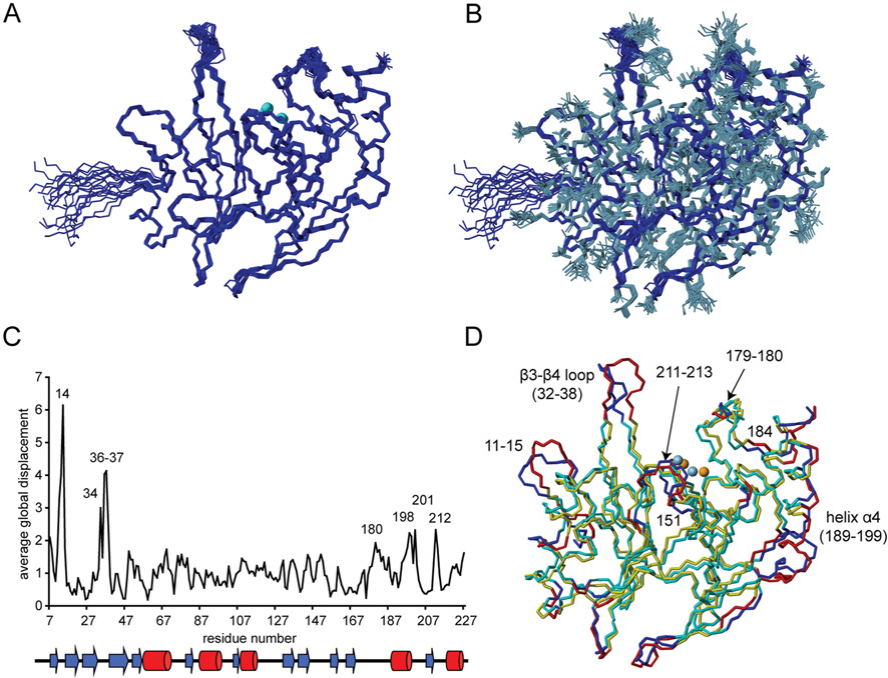
Solution structure of MBL BcII A bundle of 20 structures are shown with (**A**) only the protein backbone depicted in blue (zinc atoms in cyan) and (**B**) the amino acid side chains (heavy atoms, residues 7–227) also included in grey. (**C**) Average global displacement values comparing the BcII crystal structure (PDB code 1BVT) and the free enzyme solution structure as a function of the amino acid sequence with secondary structure elements indicated. (**D**) Comparison of the crystal structure (PDB code 1BVT) in yellow and the solution structure in cyan, the regions with RMSD>0.4 Å being highlighted in red and blue respectively. The solution structure shown is the member of the ensemble of 20 lowest energy structures closest to the mean structure (calculated with MOLMOL).

The structure of BcII shown in Figures 1(A) and 1(B) is the first solution structure reported for any MBL. As indicated by the RMSD values given in Table 1, the structure is well defined by the NMR data. The overall fold clearly corresponds to the classic MBL fold, of the four-layered αβ/βα class, with a central β-sheet sandwich flanked on either side by α-helices; the active site, with the two zinc ions, is at one edge of the β-sheet sandwich, flanked by two long loops [residues 33–38(60–66) and 170–188(223–241)]. {All residue numbers are presented as: number in BcII sequence(number in standard BBL system) [4].} The RMSD values between the member of the NMR structure ensemble closest to the mean and the 1BVT crystal structure [46], chosen for comparison since it has the highest resolution of the crystal structures reported for BcII, are 1.25 Å for the backbone and 1.96 Å for all heavy atoms, when comparing the parts of the protein which are well-defined in the NMR structure (residues 8–226). If the β3–β4 loop and residues 11–15 (see below) are excluded from the comparison, the backbone RMSD between the two structures drops to 1.00 Å. The zinc co-ordination geometry is also very similar in solution and in the crystal (Supplementary Figure S1 at http://www.biochemj.org/bj/456/bj4560397add.htm), although it should be noted that the two highest-resolution crystal structures were obtained at significantly lower pH (pH 5.2–5.6 [46,47]) than the solution structure (pH 6.4) and the nature of the anions in solution is different.

As can be seen in Figures 1(C) and 1(D), the secondary structure elements of the structure are closely similar in solution and the crystal, the regions with RMSD >0.4 Å being largely restricted to surface loops. Three of these are worthy of comment: (i) residues 11–15(36–40); (ii) the β3–β4 loop [residues 33–38(60–66)]; and (iii) the β11–α4 loop and helix α4 [residues 170–199(223–252)]. The construct used in the crystallographic study [46] was six residues shorter at the N-terminus than that used in the present study. Perhaps, as a result, residues 11–14 appear to be flexible in the 1BVT structure and were modelled with zero occupancy in the crystal, explaining the observed differences from the solution structure.

The active site of BcII is flanked by two loops, β3–β4 [residues 32–38(60–66)] and β11–α4 [residues 170–188(223–241)]. The main variability between the different crystal structures of BcII is encountered in the β3–β4 loop, the ‘mobile loop’ or ‘flap’, where all crystal structures show very poor or missing electron density. Many of the residues of this loop are either missing from the PDB files (1BC2, 2BC2, 3BC2 and 1BMC) or, in the case of 1BVT, where residues 32–38 are displayed with zero occupancy, are modelled according to other MBL structures. In the solution structure, although this surface loop has, as would be expected, a higher RMSD within the NMR structural ensemble than residues in the structural core of the protein (Figure 1), it does have a relatively well-defined conformation. This conformation is consistently seen in all the calculated NMR structures and, importantly, it is well supported by a substantial NOE network covering even residues at the tip of the loop (Supplementary Figure S2 at http://www.biochemj.org/bj/456/bj4560397add.htm); a similar well-defined NOE network has been reported for the corresponding loop of CcrA MBL, although a structure was not calculated [48]. The structure in the present study thus represents the first reliable depiction of the conformation of this functionally important loop in the uncomplexed BcII enzyme. Importantly, the conformation is significantly different from that modelled in 1BVT, displaying a twist (Figure 1 and Supplementary Figure S2) which brings the side chains of some of the loop residues (notably Phe^34^ and Val^39^) into a more central position over the active-site cavity.

The conformation of the long β11–α4 loop [residues 170–188(223–241)] on the opposite side of the active-site cleft shows modest differences between solution and crystal structure, mostly in the backbone of the highly conserved residues Gly^179^ and Asn^180^. Perhaps, as a result, there is a small displacement [0.7 Å on average for residues 189–199(242–252)] of the penultimate helix (α4) of BcII in solution as compared with the crystal structure.

### The structure of the BcII–thiomandelate complex

**Figure 2:**
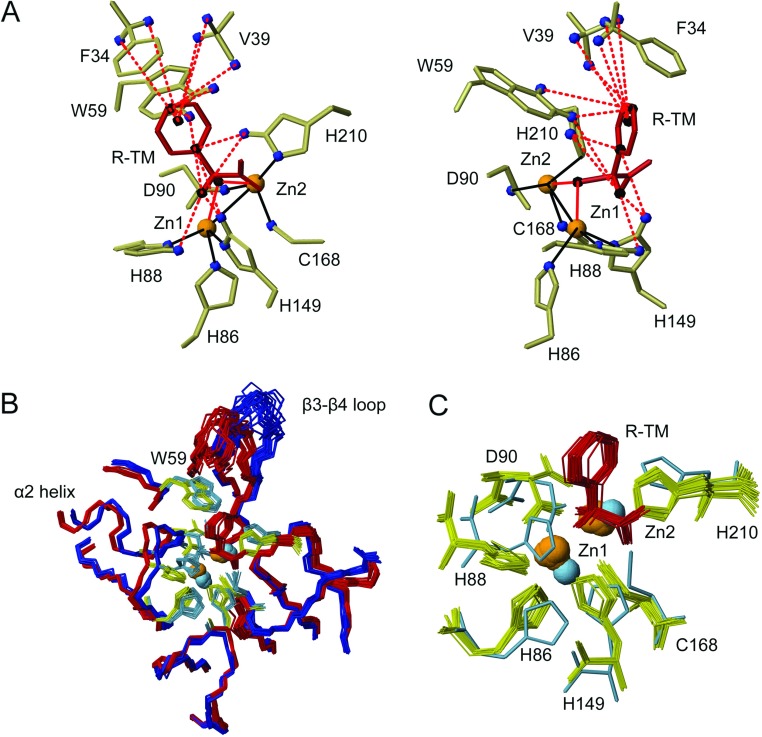
The active site of the BcII–thiomandelate complex (**A**) Two views of the lowest energy structure illustrating the constraints based on NOEs detected between the enzyme and thiomandelate. These constraints are depicted as red broken lines and the atoms and pseudoatoms involved in these constraints are shown as blue or black spheres. The inhibitor is shown in red and the zinc atoms are depicted as orange spheres. The zinc-co-ordination constraints are represented by black lines and constraints between the zinc atoms and the inhibitor sulfur atom are represented by red solid lines. (**B**) Superimposition (on residues with RMSD <0.45 Å) of the solution NMR structures (bundle of 20 lowest energy structures) of BcII (blue, side chains light blue) and the BcII–thiomandelate complex (red, side chains yellow, inhibitor red), focusing on the important active site residues. (**C**) Detail of the BcII–thiomandelate complex structure highlighting the metal co-ordinating residues and the zinc atoms (a single free enzyme structure is included as reference in light blue). (**B**) and (**C**) have the same orientation.

Information on enzyme–inhibitor contacts in the complex was obtained from detection of NOEs between protons of the enzyme and the bound inhibitor, using ^13^C-filtered NOESY experiments. The network of 13 inhibitor–enzyme NOE constraints is shown in Figure 2(A). NOEs are detected between the benzylic (Hα) proton of the inhibitor and the imidazole HE1 protons of His^88(118)^, His^149(196)^ and His^210(263)^ as well as the HH2 proton of the indole ring of Trp^59(87)^. Additionally, the *ortho* protons of the aromatic ring of the inhibitor show NOE connectivities with the HE1 protons of His^149(196)^ and His^210(263)^, and the *meta* protons with the indole HH2 and HZ2 protons of Trp^59(87)^. There are also ambiguous NOEs (assigned to the QR pseudoatom for the whole thiomandelate ring) to both methyl and C_β_ protons of Val^39(67)^ and the C_β_ protons of Phe^34(61)^. In addition we used two constraints connecting the sulfur atom of thiomandelate to each of the two zinc atoms; we demonstrated previously [27] that the thiolate group of thiomandelate co-ordinates to the two metal ions in cadmium-substituted BcII.

**Figure 3:**
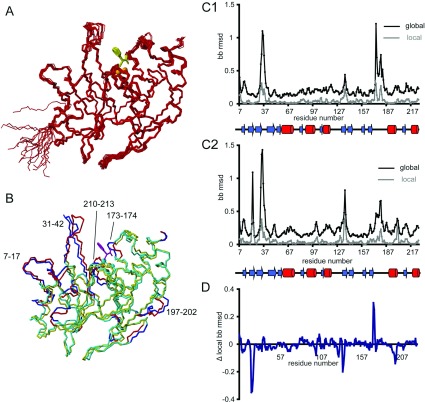
Solution structure of the BcII–thiomandelate complex (**A**) A bundle of 20 low-energy structures is shown; the protein backbone is coloured red, the zinc atoms are depicted as orange spheres and the inhibitor molecule is coloured yellow. (**B**) Comparison of the solution structures of the free enzyme (in cyan) and the complex (in yellow) with the regions with RMSD >0.45 Å highlighted in blue and red respectively. The inhibitor molecule is coloured magenta. The structures are the lowest energy structures which are additionally the closest to the mean structures. (**C**) Average global (black) and local (grey) backbone RMSDs for the BcII solution structure (**C1**) and the BcII–thiomandelate complex solution structure (**C2**). (**D**) Difference between the local backbone RMSD values in the two structures. The RMSD values are shown as a function of the amino acid sequence with the secondary structure elements indicated.

The overall structure of the enzyme–inhibitor complex (Figure 3A) is well-defined by the NMR data (RMSD for heavy atoms of 0.73 Å over residues 7–227) and is very similar to that of the free enzyme (Figure 3B). The local backbone RMSD profile (Figure 3C) of the BcII–thiomandelate complex is also similar to that of the free enzyme, with high values in the expected regions, such as the β3–β4 and the β11–α4 loops and the unstructured N-terminus (see also Supplementary Figure S3 at http://www.biochemj.org/bj/456/bj4560397add.htm). Detailed structural comparisons will focus on the active site and on the two loops which flank it.

### The BcII–thiomandelate complex: the active-site cavity and the zinc ligands

**Figure 4:**
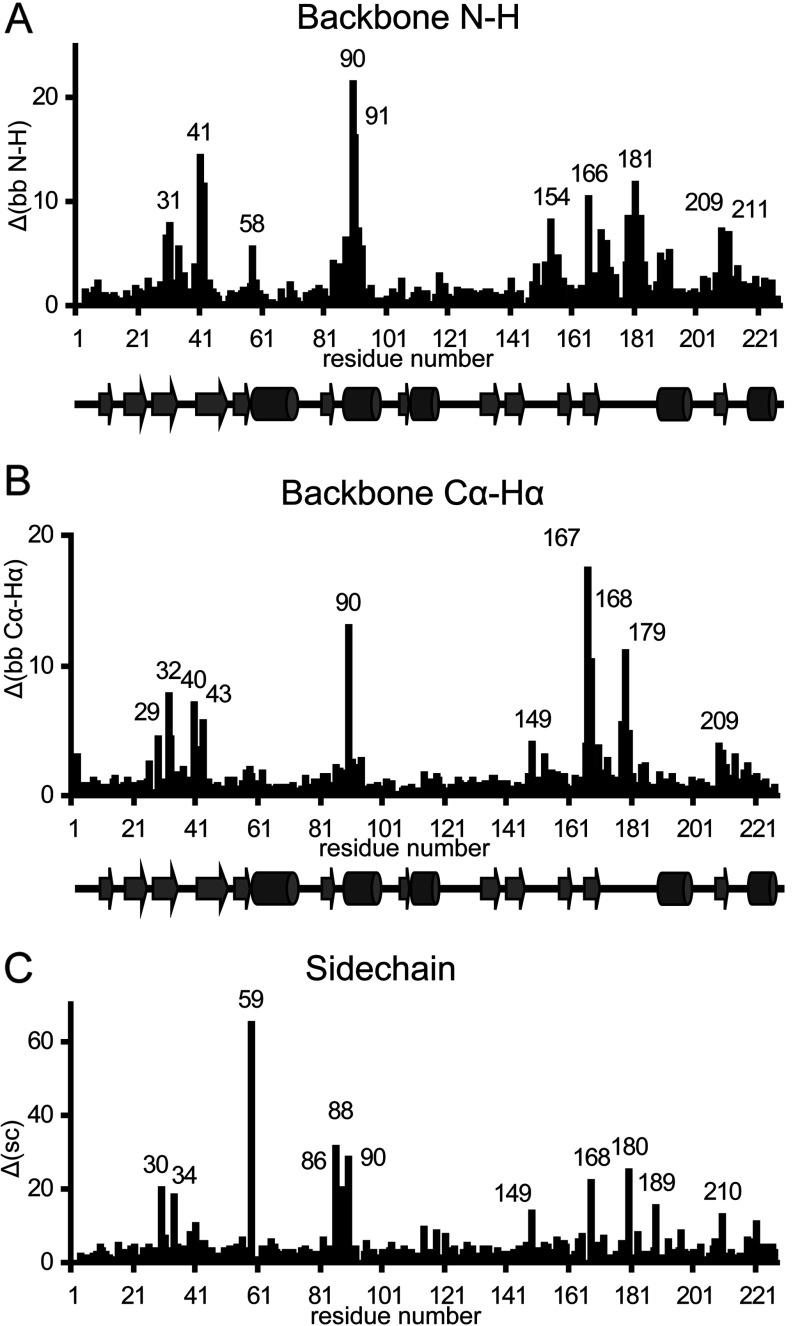
Chemical shift perturbations upon *R*-thiomandelate binding Chemical shift differences are shown as a function of the amino acid sequence, with the secondary structure elements indicated. (**A**) Backbone N-H 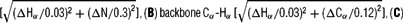 side chain 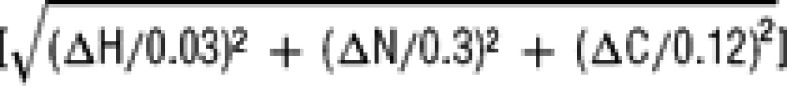.

The protein–ligand NOEs shown in Figure 2(A) define the location of the inhibitor in the active-site cavity quite precisely (Figures 2B and 2C). Comparing the free enzyme and the inhibitor complex by superimposing those parts of the two structures which have RMSD <0.45 Å, it appears that the polypeptide segment 85–91(115–121) at the end of the β6–α2 loop opens up slightly on inhibitor binding, increasing the space in the active-site cavity. Large chemical shift perturbations are observed for these residues (Figure 4), including the zinc ligands His^86(116)^, His^88(118)^ and Asp^90(120)^; the effects on Asp^90(120)^ are particularly large (Δδ ^15^N=−5.071, H_N_=+0.410, ^13^C_α_=−0.449, H_α_=+0.520, ^13^C_β_=−1.184, H_β2_=+0.669 and H_β3_=−0.107 p.p.m.). This movement of residues 85–91(115–121) is associated with a displacement of helix α2 [residues 92–101(122–131)] along its axis by approximately 1.3 Å (Figure 2B) and with changes in the side chain conformation of the three metal ligands, His^88(118)^ having a clearly different conformation (Figure 2C). This latter residue is in close proximity to the bound inhibitor, showing an NOE from its HE1 proton to the CαH of thiomandelate.

The comparison of the structures of the free enzyme and the complex shown in Figures 2(B), 2(C) and 3(B) also suggests that the position of the zinc atoms changes significantly on thiomandelate binding. Some caution must be exercised in this comparison, since constraints on zinc–ligand and zinc–zinc distances were used to locate the metal ions in the structures (see the Supplementary Online Data). However, these constraints were identical in the determination of structures of the free enzyme and the complex and were only applied during structure refinement. The NOE-based constraint set, without metal co-ordination constraints, led to well-defined positions of the zinc ligands, closely similar to those after refinement (Supplementary Figure S4 at http://www.biochemj.org/bj/456/bj4560397add.htm). This gives one confidence that the differences in zinc position shown in Figure 2 can be attributed to the changes in the NOE constraint networks of the residues involved and are likely to be real. Recent high-resolution crystallographic and molecular dynamic studies of the B1 subclass MBL NDM-1 [49] emphasize that the zinc ions have the flexibility to move significantly within the active site. The changes in position of the zinc atoms on thiomandelate binding are likely to be the result of the facts that: (i) the bound inhibitor occupies space previously occupied by zinc ligands (notably His^88^ and Asp^90^, Figure 2C), leading to changes in the position of these ligands; and (ii) the inhibitor sulfur which co-ordinates the metals in the complex has a larger radius than the oxygen of the water or hydroxide ion which co-ordinates them in the free enzyme.

### The BcII–thiomandelate complex: the β11–α4 loop

The long β11–α4 loop [168–185(221–238)], which begins with the zinc ligand Cys^168(221)^, contains some of the residues whose chemical shifts are most affected by thiomandelate binding (Figure 4). However, there is little structural change in this region of the enzyme and perturbations of side chain chemical shifts are generally larger than those of the backbone. The largest structural differences are seen for residues 173–176(226–228) and 179–180(232–233), the backbone of the loop moving somewhat further from the active-site cavity in the complex, again contributing to increasing the available space, although the differences in position between the two structures are small (≤1.2 Å). There is evidence for a decrease in flexibility of this loop in the complex (Figure 3D), notably at residues Thr^173(226)^ and Ser^174(227)^; in the free enzyme the bundle of structures is divided into two subgroups of different backbone conformation for residues 173–174 creating the high local RMSD values. The backbone amide ^1^H and ^15^N resonances of Asn^180(233)^ are not observed in the spectrum of the free enzyme, but become observable in that of the complex, again suggesting some decreased mobility on inhibitor binding.

In the crystal structures of the IMP-1 and VIM-2 MBLs with mercaptocarboxylate inhibitors [16,50], residues in this loop interact with the carboxylate group of the inhibitor; an asparagine residue equivalent to Asn^180(233)^ in BcII is seen to interact with the carboxylate in both structures, and a lysine residue equivalent to Lys^171(224)^ in BcII interacts in the IMP-1 complex (in the VIM-2 enzyme the equivalent residue is a tyrosine). In our earlier docking of thiomandelate into the crystal structure of free BcII [27], we assumed that Lys^171^ did indeed interact with the inhibitor carboxylate group. However, the structure of the complex we have now determined shows clearly that, even though the Lys^171^ side chain faces the carboxylate of the inhibitor, the distances between the two oxygen atoms and the NZ atom of Lys^171^ are too great (5.27 and 4.97 Å) to allow a direct interaction. Similarly, the side chain of Asn^180^ is relatively near the inhibitor carboxylate (6.3 Å for HB3 and 7.7 Å for HD21), but too distant for a direct interaction. The positions of these two side chains are well supported by an NOE network involving surrounding residues (see Supplementary Figure S5 at http://www.biochemj.org/bj/456/bj4560397add.htm). Importantly, the covalent structures of the mercaptocarboxylate inhibitors studied by Concha et al. [16] and Yamaguchi et al. [50] are such that the carboxylate is further from the zinc-co-ordinating thiolate group than is the case for thiomandelate. Assuming that the primary interaction is that between the inhibitor thiolate and the zinc atoms, this difference in inhibitor structure presumably accounts for the inability of the carboxylate of thiomandelate to interact directly with Lys^171^ and Asn^180^, although it is entirely possible that an interaction mediated by a bound water molecule may be present in the case of Lys^171^.

### The BcII–thiomandelate complex: the β3–β4 mobile loop and the hydrophobic pocket

The β3–β4 mobile loop or flap [residues 32–39(59–67)] of B1 MBLs has been shown to play a role in substrate and inhibitor binding, and catalysis [30]. With the exception of a glycine residue at position 63 (BBL numbering), none of the residues in the loop is fully conserved, although the residues corresponding to Phe^34(61)^ and Val^39(67)^ are hydrophobic in all B1 class MBLs. The IMP-1 and CcrA MBLs have a loop which is one residue longer, and have a tryptophan residue near the tip of the loop at position 64 (BBL numbering) which interacts with the bound ligand [14,16,17,28], but makes only a modest contribution to the binding energy of thiomandelate and another mercaptocarboxylate inhibitor [30]. In these enzymes there is a significant decrease in mobility of the loop upon inhibitor binding [48,51] and it moves towards the bound inhibitor [16]. In the BcII–thiomandelate complex, the aromatic ring of the inhibitor interacts with residues Phe^34(61)^ and Val^39(67)^ in the loop as well as with Trp^59(87)^ which is situated at the base of the loop; these residues form a conserved hydrophobic pocket which is clearly important for binding the inhibitor (Figure 2A). Chemical shifts of all the residues in the β3–β4 loop are perturbed to some degree (Figure 4). The effects, especially on backbone chemical shifts, are greatest for residues at the base of the loop, and in addition residues in the β5–α1 loop [residues 56–60(84–88)], at the base of the β3–β4 loop, are clearly affected. Indeed, Trp^59(87)^ is the most affected of all residues in terms of chemical shifts, with three protons of the indole ring showing upfield shifts of as much as 0.678 p.p.m., reflecting its proximity to the aromatic ring of thiomandelate in an edge-to-face orientation. [In IMP-1 and CcrA, Trp^59(87)^, otherwise conserved in B1 MBLs, is replaced by a phenylalanine residue which also contributes to the hydrophobic pocket involved in inhibitor binding.]

**Figure 5:**
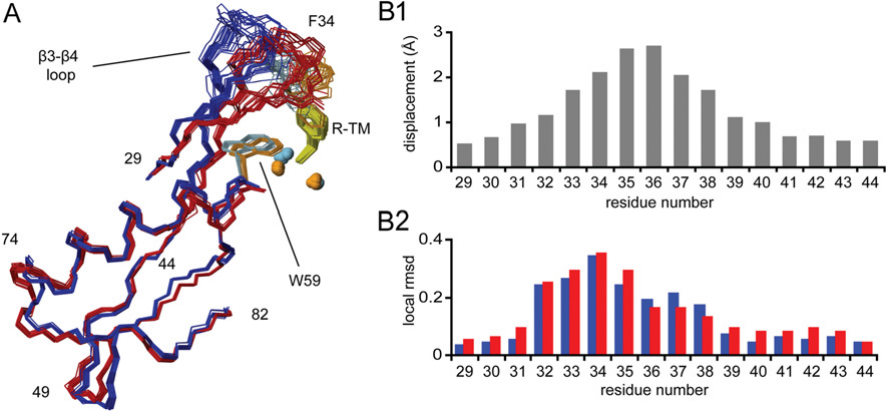
Structure and variability of the β3–β4 loop (**A**) Amino acids 29–82, encompassing the β3–β4 loop, are highlighted in the superimposition of the free enzyme (blue) and complex (red) structures. The side chains of Trp^59^ and Phe^34^ are light blue (free enzyme) or orange (complex). The inhibitor molecule is yellow and the zinc atoms are cyan (free enzyme) or orange (complex). (**B**) Backbone global displacement values (**B1**) highlighting the movement of the β3–β4 loop, and local backbone RMSDs for the two structures (**B2**; free enzyme blue and complex red) for residues 29–44 as a function of residue number.

Thiomandelate, a relatively small molecule, makes less extensive interactions with residues in the β3–β4 loop compared with many of the other inhibitors whose binding to MBLs has been studied crystallographically. However, these interactions do lead to a substantial movement (up to 2.5 Å) of the loop towards the inhibitor (Figure 5A). This movement is somewhat less than that seen in the complex of IMP-1 with a much larger mercaptocarboxylate inhibitor, where Val^25^ and Val^29^ are displaced by approximately 2.9 Å [16]. It is likely that in MBLs with a tryptophan residue at the tip of the loop (CcrA, IMP family) the interactions of this residue with the inhibitor lead to a rather greater movement of the loop than in those enzymes where this tryptophan is absent (BcII, VIM family).

As discussed above, in the free enzyme the β3–β4 loop has a quite well-defined conformation, notwithstanding the relatively large RMSD values which would be expected for a surface loop. The same is true of the BcII–inhibitor complex, where the conformation of this loop is again well-defined by an extensive network of NOEs (Supplementary Figure S2). Comparing the structures of the free enzyme and the complex in this region (Figure 5A) suggests that there is a hinge-like motion of the loop on inhibitor binding, reflected in the fact that the displacement is at a maximum at the tip of the loop (Figure 5B1), with its local conformation being largely unaffected. This motion does not appear to be accompanied by any decrease in flexibility; the local backbone RMSD of the loop residues does not change significantly upon inhibitor binding (Figure 5B2). This is in contrast with a number of crystallographic studies where, as discussed above, electron density for the β3–β4 loop is often missing in the free enzyme, but the conformation of this loop becomes well-defined in complexes with inhibitors. The fact that a significant degree of flexibility in this loop is retained in the complex, notwithstanding its direct interactions with the inhibitor, would of course still be compatible with the maintenance of a hydrophobic pocket for the aromatic ring of thiomandelate.

### Structure–activity relationships for inhibitor binding to B1 and B3 MBLs

In attempting to design improved inhibitors of MBLs which might be of clinical value, there are two distinct issues: improving affinity and retaining a broad spectrum of activity against MBLs of all classes. Thiomandelate is a potent (*K*_i_=30–500 nM) inhibitor of subclass B1 and B3 MBLs, which are di-zinc enzymes, but is a much poorer inhibitor (*K*_i_=144 μM) of the subclass B2 CphA enzyme from *Aeromonas hydrophila* [21]. The CphA enzyme functions as a mono-zinc enzyme, and is a strict carbapenemase [2]; we have shown that mercaptocarboxylate inhibitors bind quite differently to this enzyme, with the carboxylate interacting with the zinc atoms [20].

In the present study we will focus on the implications of the elucidated structures for the design of high-affinity inhibitors of subclass B1 (including the clinically important IMP, VIM and NDM enzymes) and B3 MBLs. It is clear that the interaction of the thiolate group with the zinc ions is the major determinant of the affinity of mercaptocarboxylate inhibitors for the B1 and B3 enzymes [21]. The importance of the thiolate–zinc interaction will restrict the possibility of substitutions on the inhibitor close to the thiolate; the structure of the BcII–thiomandelate complex clearly shows that the benzylic carbon (Cα) of the inhibitor is close to the zinc ligands His^88(118)^ and His^149(196)^ and substitution at this position would be unfavourable.

**Figure 6:**
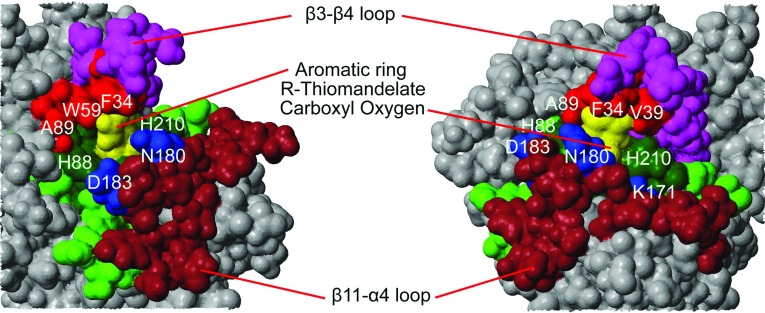
The active site of the BcII–thiomandelate complex Two views of a van der Waals surface representation of the structure of the complex are shown. The bound *R*-thiomandelic acid is coloured yellow and the interacting or potentially interacting elements of BcII are coloured as follows: the β3–β4 loop (residues 29–43) is magenta, the metal co-ordinating residues are dark green [with adjacent residues in the sequence (±2 residues) shown in green] and the β11–α4 loop (residues 171–188) is brown. Residues forming a hydrophobic pocket are coloured red and conserved residues involved in potential hydrogen-bonding contacts are coloured blue. The rest of the protein is coloured grey. The zinc atoms are buried and invisible in this representation.

As we have noted previously [20], two regions of the active-site cavity which could be exploited in inhibitor design are a conserved hydrophobic pocket and a conserved hydrogen-bonding region, shown in Figure 6. The latter comprises residues in the β11–α4 loop [see above; residues 170–188(223–241)]. This loop is shown in brown in Figure 6, with Lys^171(224)^, Asn^180(233)^ and Asp^183(236)^ conserved as hydrogen-bonding residues in B1 enzymes shown in blue; in the B3 enzymes AIM-1 and SMB-1, residue Gln^157^, in an α4–β7(156–166) loop which is unique to the B3 subclass, is positioned similarly to Asn^180(233)^ [26,52]. In B3 enzymes the loop corresponding to β11–α4 in BcII also includes serine, threonine, asparagine or tyrosine residues at positions 221, 223, 225 and 228 (BBL numbering), which may be involved in β-lactam recognition (see [26]) and thus can be targeted in inhibitor design. The carboxylate group of thiomandelate increases the affinity of the inhibitor for BcII ~100-fold [21], even though the structure shows that it is too far away for a direct interaction with Lys^171(224)^ or Asn^180(233)^. It is apparent from this and from the very potent inhibition of MBLs observed for some other mercaptocarboxylates [19,20,25,53–57] that the distance from thiolate to carboxylate is a key feature of the structure–activity relationship among these compounds. From the structure described in the present study, one or more methylene groups would need to be inserted between the benzylic carbon and the carboxylate group to allow the latter to interact with the conserved Lys^171(224)^ and/or with Asn^180(233)^ and a flexible linkage of this kind would also permit favourable interactions with the other hydrogen-bonding residues in this region in B3 enzymes.

Bulky hydrophobic groups, such as the aromatic ring of thiomandelate, forming contacts with non-polar residues on the mobile β3–β4 loop and/or hydrophobic pockets at the base of the loop, are encountered in the majority of B1 subclass MBL–inhibitor complexes whose structure has been determined [15–17,28] and are a common feature of an effective inhibitor. In Figure 6, residues of the β3–β4 loop are shown in magenta, with residues forming a hydrophobic pocket around the aromatic ring of the inhibitor shown in red. The flexibility of both the β3–β4 and the β11–α4 loops (notably the existence of an ‘adaptable’ hydrophobic pocket) undoubtedly contributes to the ability of MBLs to bind a structurally diverse range of inhibitors [30,58]. However, in the B3 enzyme the β3–β4 loop is much shorter, due to shorter β3 and β4 strands, and further away from the active site. In its place, it is the α3–β7 (in L1) or α4–β7 (in SMB-1) loop [26,59] which forms an equivalent lining to one side of the active site and contains hydrophobic residues which can interact with inhibitors [60]. The part of the aromatic ring of thiomandelate most accessible for substitution appears to be the *ortho* and *meta* positions, blocked by Phe^34(61)^ and Val^39(67)^ in the loop on one side but accessible on the other. In addition to the hydrophobic pocket formed by the β3–β4 loop in the B1 enzymes or the α3–β7/α4–β7 loop in the B3 enzymes, a number of residues in the β11–α4 loop [168–185(221–238)] with conserved hydrogen-bonding character are also suitably placed for interaction with such substituents. It is clear that, in view of the significant structural differences between B1 and B3 enzymes, for both the hydrogen-bonding region and the hydrophobic pocket, flexible substituents would be favoured for broad-spectrum activity.

### Conclusions

The structure of BcII described in the present study is the first structure of a MBL in solution. Since the distribution of NMR constraints over the sequence has no significant gaps, we can conclude that this structure is a reliable representation of the structure in solution. By contrast, in the X-ray crystallographic structures electron density was missing for some important regions, such as the β3–β4 loop, and these were modelled with zero occupancy. The conformation of the β3–β4 loop observed in the solution structure of the free enzyme is the best available representation of this key region, which is involved in substrate and inhibitor binding. This loop has significant flexibility, but overall has a well-defined conformation. Comparison with the structure of the BcII–thiomandelate complex shows that this conformation is largely maintained, but the loop undergoes a hinge-like motion bringing key hydrophobic residues into contact with the bound inhibitor. The formation of a flexible hydrophobic pocket explains the ability of MBLs to bind substrates of a wide variety of structures. The key interaction of thiomandelate with the enzyme involves co-ordination of the thiolate group of the inhibitor with the two zinc atoms. The structure of the BcII–thiomandelate complex provides valuable information on the interactions which determine the structure–activity relationships among MBL inhibitors which will be useful in guiding the development of these compounds to help counteract the threat of widespread resistance to β-lactam antibiotics.

## Online data

Supplementary data
