# Supplementary material for: Solution structures of the Bacillus cereus metallo-β-lactamase BcII and its complex with the broad spectrum inhibitor R-thiomandelic acid
Source: Biochem J. 2013 Nov 22;456(Pt 3):397–407. doi: 10.1042/BJ20131003 (PMC3898119; doi:10.1042/BJ20131003)
Supplement: Supplementary data [file bj4560397add.pdf]

BJ Biomolecules

Biochem. J. (2013) **456**, 397–407 (Printed in Great Britain) doi:10.1042/BJ20131003

online data

SUPPLEMENTARY ONLINE DATA

Solution structures of the *Bacillus cereus* metallo-β-lactamase BcII and its complex with the broad spectrum inhibitor *R*-thiomandelic acid

Andreas Ioannis KARSISIOTIS*†, Christian F. DAMBLON*‡ and Gordon C. K. ROBERTS*[1]

*The Henry Wellcome Laboratories of Structural Biology, Department of Biochemistry, University of Leicester, Leicester LE1 9HN, U.K., †The School of Pharmacy and Pharmaceutical Sciences, Biomedical Sciences Research Institute, University of Ulster, Coleraine BT52 1SA, Northern Ireland, U.K., and ‡Chimie Biologique Structurale, Institut de Chimie, Université de Liège, 4000 Liège, Belgium

EXPERIMENTAL

Incorporation of the zinc atoms and the inhibitor molecule into CYANA during structure refinement

The structure determination and automated assignment module used in the present study did not take into account the presence of the two metal atoms or, in the case of the complex, the inhibitor molecule. These were incorporated through the use of the generic linker system present in CYANA. (The NOE-based constraint sets used in refinement were obtained with automated NOE assignment without the use of the linker system.) The residue ‘ION’ from the standard library was modified to a ZN residue and accordingly the ‘METAL’ atomtypes entry in the standard library was modified to ZINC. Following the last residue of the protein sequence (Lys^{227}) and the connecting linker residue (PL228), a 12 residue linker was used to connect to the first zinc atom ZN241, comprising ten LL5-type linker residues flanked by LL2-type linker residues. An identical segment was used to connect to the second zinc atom ZN254. The length of the linker segment was chosen to be of sufficient length to ensure minimum steric interference. The effect of the presence of the linker system, with the zinc atoms both fixed and unfixed, on the CYANA target function was examined with an identical set of constraints during refinement. No significant impact was detected by comparison with a refinement run lacking the linker system. The zinc atoms were fixed to the corresponding atoms of the metal-co-ordinating residues with the use of a set of six lower (lol) and upper (upl) distance bounds (for His^{86}, His^{88}, Asp^{90}, His^{149} and Cys^{168}: lol = 2.2, upl = 2.4, and for His^{210}: lol = 2.3, upl = 2.5) loosely based on the distances observed in the di-zinc X-ray structure PDB code 1BVT; in addition, a Zn–Zn interatomic distance was included (lol = 3.65, upl = 3.85) (Table S1).

In similar fashion, the inhibitor molecule was successfully incorporated into the linker system by using a novel residue definition including a cluster of pseudoatoms to ensure correct connectivity with the linker system. The PDB file of the inhibitor was read into MOLMOL [1] and the appropriate dihedral angles were defined (LB1, CHI1, CHI2, CHI3 and LB2). The atom names and types were in accordance with the IUPAC nomenclature and the CYANA 2.1 naming conventions. The dihedral angle and the atom declarations were ordered so as to be compatible with the tree structure of the dihedral angles (SYBYL 7.2 interface to DYANA). Pseudoatoms were added for the thiomandelate aromatic ring protons (QG for HG1 and HG2, QD for HD1 and HD2, and QR for all the ring protons). The creation of the overlap atoms at the beginning and end of the residue definition (corresponding to N, CA and C in the case of amino acids) which ensure the connectivity of the new residue with the generic linker residues was essential since thiomandelate is not an amino acid derivative. A connecting system such as the one used in the creation of proxy residues [2] was used successfully.

Structure calculation and refinement

Simulated annealing

It has been reported, in the case of structural calculations performed with the program ARIA, that an increase in the number of simulated annealing steps can prove to be highly beneficial in achieving convergence, especially large proteins where chemical shift degeneracy is a significant issue [3,4]. In the present study we found that the use of extended torsion angle dynamics (TAD) steps (15 times the normal) can indeed be beneficial in terms of convergence and target function values. However, these calculations tended to become biased towards similar conformations, as reflected by the unusually low RMSD values observed even in some regions expected to be unstructured. This suggests that extended TAD steps can be valuable, providing converged and essentially correct structures and reducing exhaustive peak list analysis, but must be used with caution.

Dihedral angle constraints

We found that too general a use of dihedral angle constraints derived from the TALOS database may introduce erroneous features in regions of unusual or complex backbone structure distribution, for example around the zinc co-ordination sites. Consequently, the structures reported in the present study were calculated both with and without dihedral angle constraints and we focus on the structures obtained without these constraints. Table 1 in the main text gives the structural statistics for the structures calculated without dihedral angle constraints. Table S2 gives these statistics for structures calculated with these constraints. The structures calculated with and without dihedral angle constraints had very similar RMSD values (0.35–0.38 for backbone atoms) and similar Ramachandran statistics (>98 % of residues in the core and allowed regions), but the structures calculated with dihedral angle constraints showed significantly more distance and angle violations.

[1] To whom correspondence should be addressed (email gcr@le.ac.uk).

The complete ^{1}H, ^{13}C and ^{15}N chemical shifts and NMR restraints have been deposited in the BioMagResBank (BMRB) under accession codes 19047 (BcII) and 19048 (BcII–*R*-thiomandelic acid) and co-ordinates have been deposited in the PDB under accession codes 2M5C (BcII) and 2M5D (BcII–*R*-thiomandelic acid).

© 2013 The Author(s)
The author(s) has paid for this article to be freely available under the terms of the Creative Commons Attribution Licence (CC-BY) (http://creativecommons.org/licenses/by/3.0/) which permits unrestricted use, distribution and reproduction in any medium, provided the original work is properly cited.

Biochemical Journal www.biochemj.org

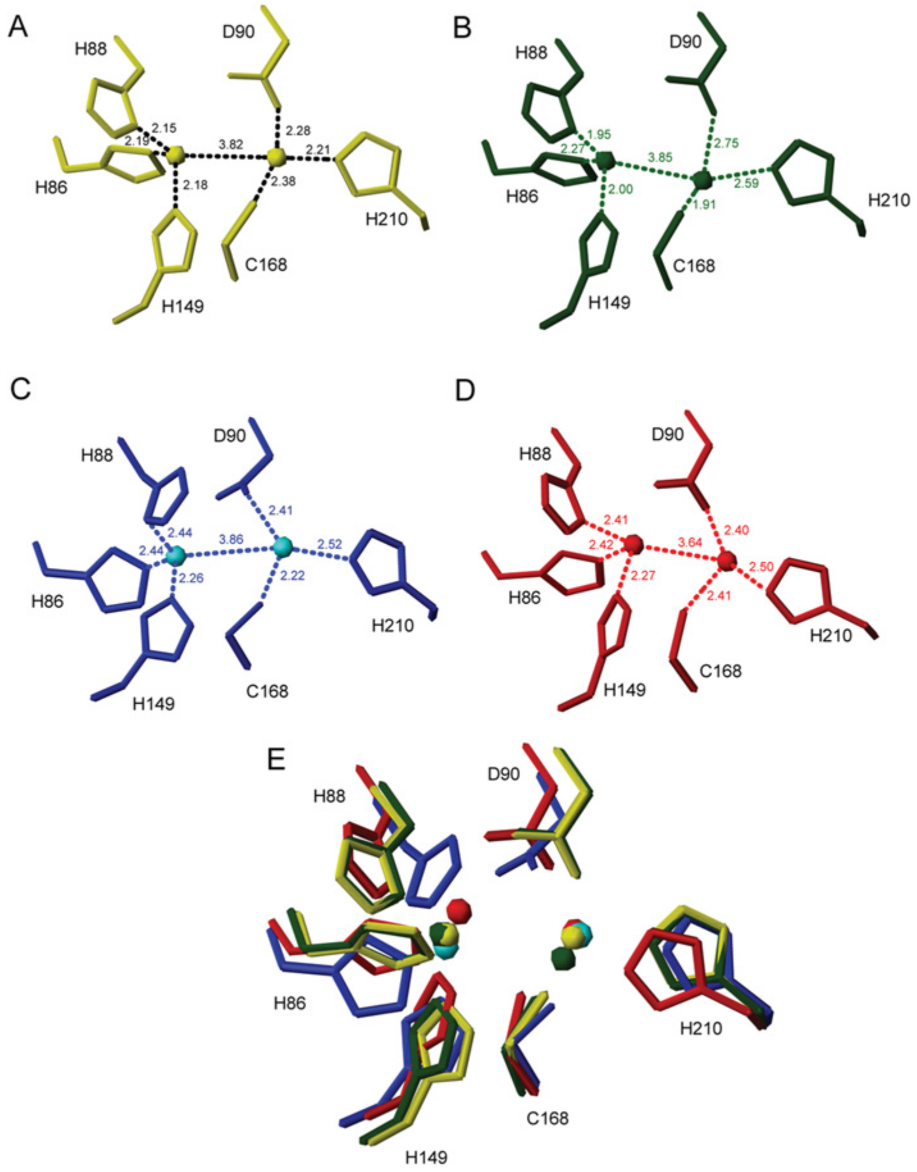

Figure S1 Comparison of zinc co-ordination in solution and in crystal

The co-ordination of the two zinc atoms by protein ligands in BcII in the free enzyme in crystal (**A**, PDB code 1BVT; **B**, PDB code 1BC2) and in solution (**C**) and in the BcII–thiomandelate complex in solution (**D**) are shown separately (**A**–**D**) and superimposed (**E**).

© 2013 The Author(s)
The author(s) has paid for this article to be freely available under the terms of the Creative Commons Attribution Licence (CC-BY) (http://creativecommons.org/licenses/by/3.0/) which permits unrestricted use, distribution and reproduction in any medium, provided the original work is properly cited.

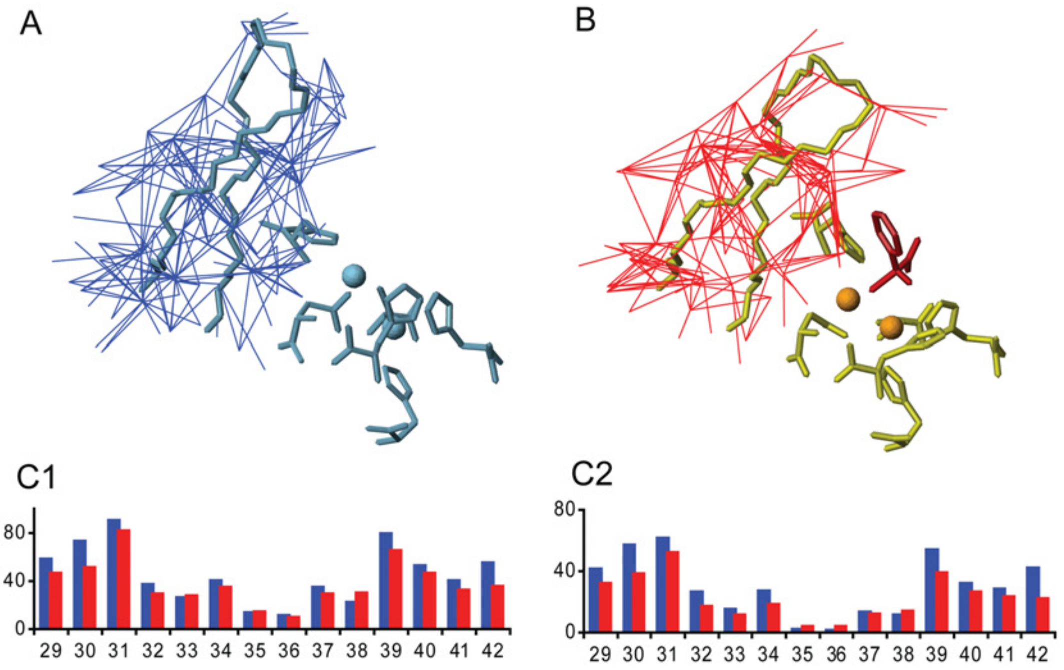

Figure S2 Constraints defining the conformation of the β3–β4 loop

The NOE constraints, including both those involving side chain atoms and those involving backbone atoms, are shown on backbone representations of the loop (residues 29–42) for (**A**) the free enzyme and (**B**) the *R*-thiomandelate complex. The two panels share the same orientation and the inhibitor molecule is presented in red. (**C**) Constraint distribution for residues 29–42, showing total number of constraints (**C1**) and long plus medium range constraints (**C2**); free enzyme constraints are shown in blue, complex constraints in red.

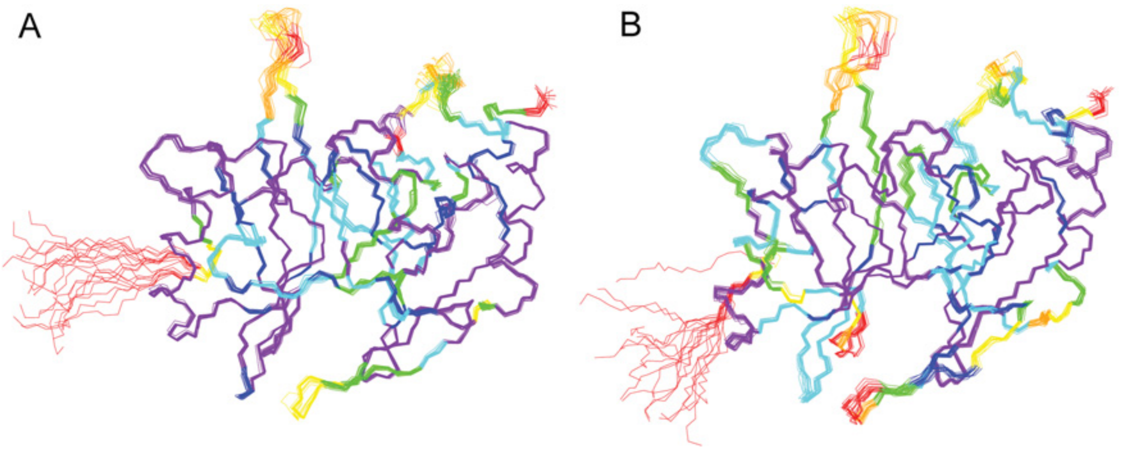

Figure S3 Local backbone RMSDs

Local backbone RMSDs are shown on the solution structures of the free enzyme (**A**) and the complex (**B**) in rainbow colours: violet (⩽0.03 Å), blue (0.03–0.04 Å), cyan (0.05–0.06 Å), green (0.07–0.09 Å), yellow (0.1–0.2 Å), orange (0.2–0.3 Å) and red (>0.3 Å).

© 2013 The Author(s)
The author(s) has paid for this article to be freely available under the terms of the Creative Commons Attribution Licence (CC-BY) (http://creativecommons.org/licenses/by/3.0/) which permits unrestricted use, distribution and reproduction in any medium, provided the original work is properly cited.

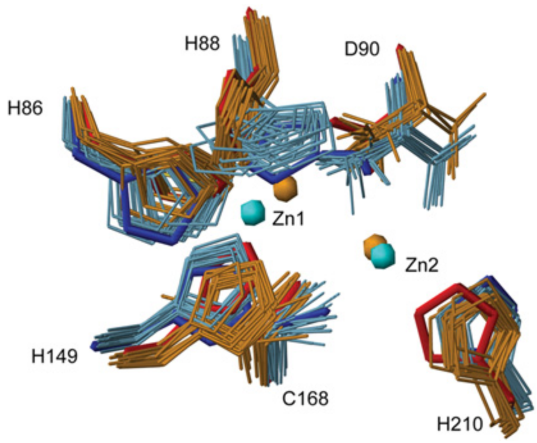

Figure S4 Zinc atoms and their protein ligands in the structures of the free enzyme and the *R*-thiomandelate complex

The bundles of structures shown were determined solely from the NOE-based constraints, before the introduction of zinc restraints in refinement. Structures are shown for the free enzyme (slate blue) and the complex (orange). The positions of the protein ligands after refinement are shown in blue (free enzyme) and red (complex) and the positions of the zinc atoms as blue (free enzyme) and orange (complex) spheres.

Table S1 Metal co-ordination constraints used (lower and upper limits) and corresponding distances on the X-ray structure (PDB code 1BVT) and both NMR structures

Bundles of 20 NMR structures were used for the distance calculations. RTM, *R*-thiomandelate.

Distance	lol	upl	1BVT	BcII	BcII–RTM
His^{86} NE2–Zn1	2.20	2.40	2.33	2.40–2.46	2.42–2.47
His^{88} ND1–Zn1	2.20	2.40	2.24	2.40–2.48	2.18–2.43
His^{149} NE2–Zn1	2.20	2.40	2.32	2.16–2.40	2.27–2.42
Cys^{168} SG–Zn2	2.20	2.40	2.29	2.19–2.41	2.24–2.44
His^{210} NE2–Zn2	2.30	2.50	2.41	2.50–2.53	2.29–2.51
Asp^{90} OD2–Zn2	2.20	2.40	2.29	2.23–2.43	2.19–2.43
Zn1–Zn2	3.65	3.85	3.75	3.82–3.88	3.62–3.65

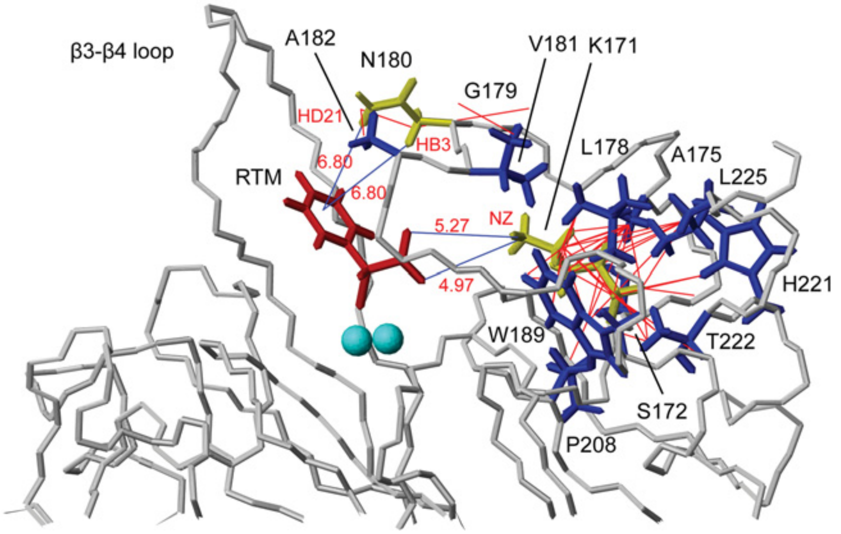

Figure S5 Conformations of Lys^{171} and Asn^{180} in the BcII–thiomandelate complex

The side chains of Lys^{171} and Asn^{180} are shown in yellow and thiomandelate in red. The constraint network defining the positions of Lys^{171} and Asn^{180} is depicted with red lines and the side chains of the residues involved in these constraints are shown in blue. The distances between the NZ atom of Lys^{171} and the oxygen atoms of the carboxylate group of the inhibitor and the distances between the HD21 and HB3 atoms of the Asn^{180} and the QR pseudo atom of the aromatic ring of the inhibitor are indicated. The zinc atoms are shown as cyan spheres and the protein backbone is coloured grey.

© 2013 The Author(s)
The author(s) has paid for this article to be freely available under the terms of the Creative Commons Attribution Licence (CC-BY) (http://creativecommons.org/licenses/by/3.0/) which permits unrestricted use, distribution and reproduction in any medium, provided the original work is properly cited.

Table S2 Structural statistics and agreement with experimental data for the refined BcII and BcII–*R*-thiomandelate structures calculated with external (TALOS) dihedral angle input

Target function, RMSD and energy are calculated for a bundle of 20 structures unless specified otherwise. RTM, *R*-thiomandelate.

Parameter	Statistics	BcII	BcII–*R*-thiomandelate
CYANA/CANDID	Input peaks	13 088	12 499
	Unassigned peaks	1082	1159
	Assignment percentage	91.7	90.7
	Target function (cycle1)	233.87	195.59
	Target function (final)	5.39	3.81
RMSD, Å (residues 7–227) cycle1	Backbone/heavy atoms	0.85/1.30	0.95/1.39
RMSD, Å (residues 7–227) final	Backbone/heavy atoms	0.30/0.66	0.31/0.67
NOE constraints	Upper distance limits	7165	6565
	Short range ($i = 1$)	3004	2815
	Medium range ($1 < i \leqslant 4$)	1257	1098
	Long range ($i > 4$)	2904	2652
	Removed during refinement	104	2
Other constraints	Non-NOE constraints (zinc)	7	9
	RTM constraints	–	13
	Dihedral restraints	206	202
	Hydrogen bonds	0	50
	Stereospecific assignments	142	111
Target function (refinement)	Minimum	2.52	2.97
	Average (20 structures)	3.1 ± 0.3	3.3 ± 0.2
	Average (50 structures)	4.3 ± 1.6	3.5 ± 0.2
RMSDs of experimental restraints (20 structures)	Upls	0.006 ± 0.001	0.006 ± 0.0003
	Lols	0.002 ± 0.002	0.005 ± 0.001
	Torsion angles	0.23 ± 0.06	0.21 ± 0.03
Van der Waals energy sums	20 structures	14.3 ± 0.7	14.1 ± 0.7
	50 structures	16.19 ± 2.34	14.88 ± 0.82
Mean violations (20/50 structures)	Distance > 0.5 Å	$0.3 \pm 0.4/0.6 \pm 0.7$	0/0
	Angle $> 5°$	$0.05 \pm 0.22/1.0 \pm 2.8$	0/0
	Van der Waals	0/0	0/0
RMSD (residues 7–227) final structures	Backbone/heavy atoms	0.38/0.72	0.38/0.72
Ramachandran statistics (CANDID stage/refinement)	Core (%)	78.6/78.7	78.2/76.6
	Allowed (%)	20.9/20.0	21.3/22.0
	Generously allowed (%)	0.1/0.6	0.1/0.8
	Disallowed (%)	0.5/0.6	0.5/0.6

REFERENCES

1 Koradi, R., Billeter, M. and Wuthrich, K. (1996) MOLMOL: a program for display and analysis of macromolecular structures. J. Mol. Graphics **14**, 51–55

2 Ab, E., Pugh, D. J. R., Kaptein, R., Boelens, R. and Bonvin, A. (2006) Direct use of unassigned resonances in NMR structure calculations with proxy residues. J. Am. Chem. Soc. **128**, 7566–7571

3 Fossi, M., Linge, J., Labudde, D., Leitner, D., Nilges, M. and Oschkinat, H. (2005) Influence of chemical shift tolerances on NMR structure calculations using ARIA protocols for assigning NOE data. J. Biomol. NMR **31**, 21–34

4 Fossi, M., Oschkinat, H., Nilges, M. and Ball, L. J. (2005) Quantitative study of the effects of chemical shift tolerances and rates of SA cooling on structure calculation from automatically assigned NOE data. J. Magn. Reson. **175**, 92–102

Received 30 July 2013/17 September 2013; accepted 24 September 2013
Published as BJ Immediate Publication 24 September 2013, doi:10.1042/BJ20131003

© 2013 The Author(s)
The author(s) has paid for this article to be freely available under the terms of the Creative Commons Attribution Licence (CC-BY) (http://creativecommons.org/licenses/by/3.0/) which permits unrestricted use, distribution and reproduction in any medium, provided the original work is properly cited.
